# Stress management and adherence among dialysis patients: A Pilot Study

**DOI:** 10.1192/j.eurpsy.2026.11027

**Published:** 2026-07-31

**Authors:** O. Impis, E. Grapsa, A. Zartaloudi, G. Gerogianni

**Affiliations:** 1 Department of Nursing, University of West Attica; 2 Medical School, Professor, University of Athens; 3 Department of Nursing, Associate Professor, University of West Attica; 4 Department of Nursing, Associate Professor, University of West Attica, Athens, Greece

## Abstract

**Introduction:**

Stress management is a predictive factor of adherence to hemodialysis regimen.

**Objectives:**

The aim of this study was to explore the association of stress management and adherence to hemodialysis regimen among hemodialysis patients.

**Methods:**

In this pilot study, forty-seven (n=47) patients on hemodialysis completed the questionnaires: i) Brief Cope for the assessment of stress management ii) GR Simplified Medication Adherence Questionnaire (GR-SMAQ-HD) for the assessment of adherence iii) A questionnaire about demographic characteristics. Spearman correlation coefficients (rho) were used to explore the association of two continuous variables. Multiple linear regression, in a stepwise method, was used to find the factors associated with adherence. Analyses were conducted using SPSS statistical software (version 26.0).

**Results:**

The results of this pilot study showed that greater behavioral disengagement was associated with lower overall adherence to hemodialysis regimen (p<<0,001). Additionally, higher substance use was significantly associated with reduced overall adherence (p<0,029). Furthermore, increased expression of negative emotions was linked to lower adherence to medications (p<<0,001), diet/fluid restrictions(p<<0,001), and overall adherence (p<<0,001).

**Conclusions:**

The use of positive thinking could be recommended to improve adherence among hemodialysis patients, since it can emphasize positive aspects of patients’ lives and training strategies for improvement their physical and mental capabilities.

**Disclosure of Interest:**

None Declared

